# Development of an individualized risk calculator of treatment resistance in patients with first-episode psychosis (TRipCal) using automated machine learning: a 12-year follow-up study with clozapine prescription as a proxy indicator

**DOI:** 10.1038/s41398-024-02754-w

**Published:** 2024-01-22

**Authors:** Ting Yat Wong, Hao Luo, Jennifer Tang, Tyler M. Moore, Ruben C. Gur, Yi Nam Suen, Christy Lai Ming Hui, Edwin Ho Ming Lee, Wing Chung Chang, Wai Ching Yan, Eileena Chui, Lap Tak Poon, Alison Lo, Koi-Man Cheung, Chui Kwan Kan, Eric Yu Hai Chen, Sherry Kit Wa Chan

**Affiliations:** 1 Department of Psychiatry, School of Clinical Medicine, Li Ka Shing Faculty of Medicine, The University of Hong Kong, Hong Kong SAR, China; 2 Department of Psychology, Education University of Hong Kong, Hong Kong SAR, China; 3 Department of Psychiatry, University of Pennsylvania, Philadelphia, PA USA; 4 Department of Social Work and Social Administration, Faculty of Social Sciences, The University of Hong Kong, Hong Kong SAR, China; 5 Department of Educational Psychology, The Chinese University of Hong Kong, Hong Kong SAR, China; 6 Department of Psychiatry, Kowloon Hospital, Hong Kong SAR, China; 7 Department of Psychiatry, Queen Mary Hospital, Hong Kong SAR, China; 8 Department of Psychiatry, United Christian Hospital, Hong Kong SAR, China; 9 Kwai Chung Hospital, Hong Kong SAR, China; 10 Castle Peak Hospital, Hong Kong SAR, China; 11 Department of Psychiatry, Pamela Youde Nethersole Eastern Hospital, Hong Kong SAR, China; 12 The State Key Laboratory of Brain and Cognitive Sciences, The University of Hong Kong, Hong Kong SAR, China

**Keywords:** Schizophrenia, Prognostic markers

## Abstract

About 15–40% of patients with schizophrenia are treatment resistance (TR) and require clozapine. Identifying individuals who have higher risk of development of TR early in the course of illness is important to provide personalized intervention. A total of 1400 patients with FEP enrolled in the early intervention for psychosis service or receiving the standard psychiatric service between July 1, 1998, and June 30, 2003, for the first time were included. Clozapine prescriptions until June 2015, as a proxy of TR, were obtained. Premorbid information, baseline characteristics, and monthly clinical information were retrieved systematically from the electronic clinical management system (CMS). Training and testing samples were established with random subsampling. An automated machine learning (autoML) approach was used to optimize the ML algorithm and hyperparameters selection to establish four probabilistic classification models (baseline, 12-month, 24-month, and 36-month information) of TR development. This study found 191 FEP patients (13.7%) who had ever been prescribed clozapine over the follow-up periods. The ML pipelines identified with autoML had an area under the receiver operating characteristic curve ranging from 0.676 (baseline information) to 0.774 (36-month information) in predicting future TR. Features of baseline information, including schizophrenia diagnosis and age of onset, and longitudinal clinical information including symptoms variability, relapse, and use of antipsychotics and anticholinergic medications were important predictors and were included in the risk calculator. The risk calculator for future TR development in FEP patients (TRipCal) developed in this study could support the continuous development of data-driven clinical tools to assist personalized interventions to prevent or postpone TR development in the early course of illness and reduce delay in clozapine initiation.

## Introduction

About 15–40% of patients with schizophrenia are considered to have treatment-resistant schizophrenia (TRS) [1–3] and were found to have 3- to 11-fold higher direct healthcare costs [4, 5], as well as poorer functional outcomes [1, 6]. Clozapine is among the most effective antipsychotics for TRS patients [7] and is considered the first-line pharmacological treatment for TRS in many countries [8]. Despite its efficacy, there are often years of delays in clozapine initiation with multiple antipsychotic trials prior to the clozapine initiation [9, 10], which was found to be related to poor response to clozapine [1, 11]. Identification of patients who are at higher risk of developing treatment resistance (TR) may reduce the delay of clozapine initiation. Though about 22% of patients are considered to be TR in their first-episode of illness [12], which is likely to have distinctly different mechanisms than those who develop TR after multiple episodes [13], the median time of TR development is up to 10 years [14, 15]. Dopamine hypersensitivity has been suggested as a possible mechanism in the development of TR [16]. Therefore, identification of individuals who have higher risk of developing TR, particularly in the early stage of the illness, would be the first step to facilitate personalized and targeted interventions to prevent or postpone the development of TR.

Though multiple factors have been explored in prospective studies as possible predictors of TRS, only 12 studies have been identified in a recent systematic review and found early age of onset as the most consistent predictor reported [17, 18]. About half of the included studies had five years or less follow-up period. Use of integrated prediction models in TRS prediction has been advocated [19]. However, there are only four studies attempting to develop a prognostic prediction model to predict TR development using machine learning (ML) methods [20–23] with three being in patients with first-episode psychosis (FEP) [20, 21, 23]. Most studies used LASSO logistic regression or forced-entry models with area under curve (AUC) ranging from 0.59 [21] to 0.67 [23]. These studies are initial attempts to establish a predictive model using ML approaches, and results suggest that more advanced ML models may be needed to improve prediction performance. Most of these studies had a moderate follow-up period (<5 years) that might have restricted the predictive performance of the established model. Furthermore, these studies and other general studies on the predictors of TRS, only included demographics and baseline information without considering treatment outcomes and clinical characteristics during the early stage of the illness, which have been related to the development of TR [1, 17]. With few previous studies, it is difficult to determine the optimal ML model to be used. Therefore, to develop a data-assisted clinical tool to estimate individual risks of TRS development, a larger pool of state-of-the-art ML models should be considered. Automated machine learning (autoML) is a process that automates the tasks of applying machine learning, including optimizing algorithm selection and hyperparameter optimization, to maximize the predictive performance of the model.

Clozapine prescription is only recommended for TRS patients in most countries and regions, including Hong Kong [8], and has been considered a proxy for TR status in many population-based studies [21, 24]. Therefore, the current study used clozapine initiation as a proxy of treatment resistance status. The aims of the current study are to establish a prediction model of future clozapine use, a proxy of TR development, among the FEP population over 12–17 years of follow-up using clinical information at baseline and over the initial three years of the treatment with autoML. Prediction models with baseline, 12-month, 24-month, and 36-month information were established separately. An individualized risk calculator for treatment resistance development of FEP patients (TRipCal) was established using the significant features identified with the autoML model. Results of the current study may provide support to the development of personalized interventions in improving outcomes of patients with FEP.

## Methods

### Data source and study sample

The sample of this study was originally included in a study comparing three-year outcomes of patients with first-episode psychosis (FEP) who were treated by early intervention services (EIS) for psychosis and those who received standard care services (SCS) [25]. A total of 700 FEP patients who were consecutively enrolled in the EIS [26] between July 1, 2001 and June 30, 2003 in all public psychiatric units in Hong Kong and age, gender and diagnosis-matched FEP patients (*n* = 700) who received the SCS between July 1, 1998 and June 30, 2001 provided by the Hospital Authority (HA) in Hong Kong were included. Patients with diagnosis of substance-induced psychosis, organic disorders, or intellectual disability, and those who had received prior antipsychotic medication for more than a month were excluded from the initial case identification. Detailed medication history of all patients (*N* = 1400) from their first service visit (EIS or SCS) to June 2015 (follow-up period: 12–17 years) were retrieved from the centralized electronic hospital database (Clinical Management System [CMS]). After excluding 2 patients with missing data for clozapine use, we identified 191 out of 1398 patients (13.7%) who have ever received clozapine prescriptions during this follow-up period. The CMS is an electronic clinical record system of the HA in Hong Kong which covers over 90% of the psychiatric care of severe mental illness patients [27]. All inpatient and outpatient clinical information including hospitalization, consultation records, medication prescription were included in the CMS. Institutional ethical approval was obtained from all Hong Kong hospital clusters for the current study. Data analysis and development of the calculator was conducted between December 2022 and March 2023.

### Outcomes and features

Clozapine use was considered as a proxy indicator of TR and the outcome in the current study. All features were obtained from case notes of each enrolled patient using a standardized CMS data entry form [28]. Features of interest at baseline included age at first service contact to the EIS or SCS, sex, years of education, any life events prior to the service entry, smoking status, diagnoses, age of illness onset, received EIS or not, duration of first episode, length of hospitalization at first-episode, duration of untreated psychosis (DUP), suicidal attempts (SA), non-suicidal self-injury (NSSI) during DUP and presence of psychiatric comorbidities. Furthermore, the clinical notes of patients were examined to summarize monthly clinical features including symptoms, functioning, other clinical information, and medication use for the first three years of clinical services. Symptom features included positive and negative psychotic symptoms assessed by the Clinical Global Impressions-Schizophrenia (CGI-SCH) scale [29] and depressive symptoms measured by the Clinical Global Impression scale (CGIS) [30]. Social functioning of patients was assessed by the Social and Occupational Functioning Assessment Scale (SOFAS) [31]. These variables were further summarized into mean and mean of the squared successive differences (MSSD) [32]. Other clinical information included SA, NSSI, substance abuse, Accident & Emergency visit, out patient departments visit, hospitalization, default from outpatient appointments, and relapse. Medication and intervention features included daily defined dose (DDD) of antipsychotic medication [33], and whether anticholinergic, antidepressant, benzodiazepine, mood stabilizer, or electroconvulsive therapy were prescribed. Information on types of antipsychotics and daily dose were used for DDD calculation and monthly average DDD of antipsychotics were determined. Operational definition of the features and the quality assurance of the data including interrater reliability are in the supplementary documents.

Model development and validation followed the guidelines of Transparent Reporting of a multivariable prediction model for Individual Prognosis Or Diagnosis (TRIPOD, Table S1). An automated machine learning (autoML) approach was implemented to automate the selection of ML algorithms and hyperparameters using the Tree-based Pipeline Optimization Tool (TPOT) package in Python [34, 35]. TPOT optimizes the search process for prediction models through employing genetic programming and evolutionary algorithms (see http://epistasislab.github.io/tpot/ for more details).

**Fig. 1:**
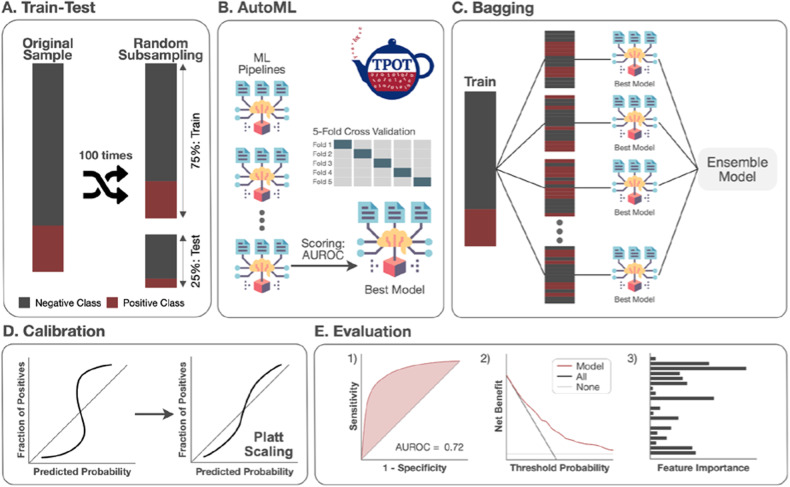
Overview of probabilistic classification model workflow. **A** We split our data using random subsampling. The former approach was the main analysis of the current study. We randomly split the participants into train (75%) and test data sets and repeat this procedure 100 times to obtain a stable performance. The latter approach aims to examine the generalization of our models. **B** AutoML was implemented in Python using the TPOT package. **C** Bagging procedure is added when re-training the best model from autoML. **D** Calibration was performed using Platt scaling. **E** Evaluation of the performance of test data includes area under the receiver operating characteristic curve (AUROC), decision curve analysis and feature importance.

To develop the probabilistic classification models, the sampling process was stratified based on the outcome variable (i.e., clozapine use) with the sample being randomly divided into training (75%) and testing (25%) sets (Fig. 1A). Missing data were imputed using median replace and the features were standardized by subtracting the mean and scaling to the unit variance in the training data. The derived preprocessing steps were then applied to test data. TPOT was set to run for 10 generations with a population size of 50 pipelines (Fig. 1B). For each generation, the best model was selected based on the area under the receiver operating characteristic (AUROC), evaluated through a 5-fold cross-validation (CV) within the training data. The training data was re-fitted with the best model plus bagging and calibration procedures (Fig. 1C, D) to minimize overfitting and improve out-of-sample model performance. To obtain a stable performance and avoid overfitting to a particular subsample, we repeated the above procedure 100 times. For each repeat, the AUROC, calibration performance measured by Brier score, decision curve analysis and feature importance were calculated (Fig. 1E). Detailed approaches of decision curve analysis are in the supplementary methods.

The overall model performance was obtained by averaging AUROC and Brier scores across 100 repetitions. We ranked the feature importance for each repetition as different algorithms may be selected for each repetition. In scikit-learn, feature importance represents the relative importance of each feature in a trained model for predicting a target variable. Average feature importance rank for each variable was calculated across 100 repetitions for further interpretation. Four probabilistic classification models were developed by incorporating features of different duration (i.e., baseline or first month, 12, 24 and 36-month). For each model, we removed patients with clozapine use within the period of the features (Baseline *n* = 1398; 12-month *n* = 1387; 24-month *n* = 1379; 36-month *n* = 1363).

Finally, the features were reduced to a reasonable number by refitting the data using the above procedures with top 10, 15 or 20 features and comparing their performance to determine an optimal number of features. A risk calculator was developed with the optimal number of features to calculate predicted probabilities of future clozapine use of FEP patients.

## Results

### Sample characteristics

Table 1 displays the comparison of basic demographics between patients with and without clozapine use. In general, patients with clozapine use compared to their counterparts had younger age of first service contact, with a lower education level, were more likely to have a schizophrenia diagnosis, and younger age of illness onset. The mean duration of first use of clozapine from the first service contact was 83.9 months (7 years) (SD = 48.9, median = 76.7, range = [2.17, 201.2]).

**Table 1:** Sample characteristics of first-episode psychosis (FEP) patients with clozapine use compared to those without.

	Clozapine Use				
Characteristic	No, *N* = 1207^a^	Yes, *N* = 191^a^	T/${\chi }^{2}$	*p*-value^b^	*q*-value^c^
Age at first service contact	21.91 (3.41)	20.27 (3.30)	6.4	<0.001	**<0.001**
Sex			0.10	0.8	0.8
Male	617 (51%)	100 (52%)			
Female	590 (49%)	91 (48%)			
Years of education	10.82 (2.39)	10.14 (2.19)	3.9	<0.001	**<0.001**
Diagnosis			39	<0.001	**<0.001**
Schizophrenia	775 (64%)	166 (87%)			
Other diagnoses of psychotic disorders	432 (36%)	25 (13%)			
Age of illness onset	20.76 (3.47)	19.26 (3.38)	5.7	<0.001	**<0.001**
Treatment			3.8	0.052	0.072
Early Intervention	616 (51%)	83 (43%)			
Standard Care	591 (49%)	108 (57%)			
DUP days (log)	4.13 (1.87)	4.25 (1.60)	−0.93	0.4	0.4

^a^Mean (SD); *n* (%).

^b^Welch Two Sample *t*-test; Pearson’s Chi-squared test.

^c^False discovery rate correction for multiple testing.

The bold values are significant value after the false discovery rate correction for multiple testing.

### Probability classification and predicted probability

**Fig. 2:**
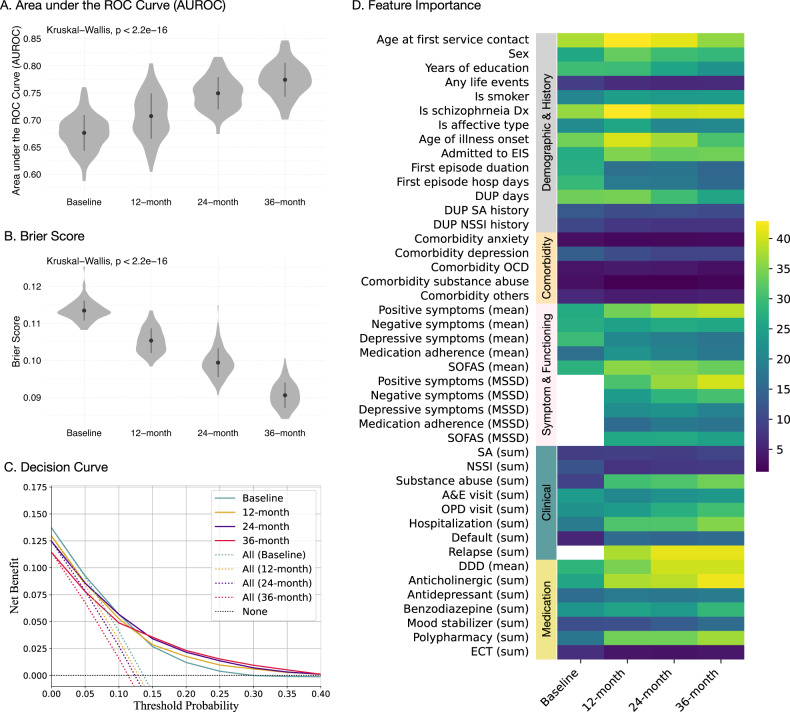
Performance of the baseline, 12-, 24- and 36-month probability classification models. **A** Area under the receiver operating characteristic curve (AUROC). **B** Brier score. **C** Decision curve. The results indicate that models with longer longitudinal information performed better. **D** The average feature importance rank was calculated across 100 repetitions of the autoML procedures, where a higher value indicates greater importance of a feature. Dx diagnosis, EIS early intervention service, DUP duration of untreated psychosis, SA suicide attempts, NSSI non-suicidal self-injury, OCD obsessive-compulsive disorder, SOFAS Social and Occupational Functioning Assessment Scale, A&E accident & emergency, OPD out patient departments, DDD daily defined dose, ECT electroconvulsive therapy.

Figure 2A shows distribution of the AUROC with a mean and standard deviation (SD) over 100 repeated random subsampling. The autoML model discriminated between patients with and without future clozapine use with a baseline AUROC of 0.676 (SD = 0.033, 95% CI = [0.670, 0.683]), a 12-month AUROC of 0.707 (SD = 0.042, 95% CI = [0.699, 0.716]), a 24-month AUROC of 0.749 (SD = 0.030, 95% CI = [0.744, 0.755]), and a 36-month AUROC of 0.774 (SD = 0.031, 95% CI = [0.768, 0.780]). The model with longer longitudinal information had a better discrimination ability (Kruskal–Wallis *χ*^2^ = 227.9, df = 3, *p* < 2.2e-16).

Figure 2B shows that each model had a low Brier score (<0.12), suggesting moderate to good agreement between observed and expected risk. The Brier scores were 0.113 (SD = 0.0027, 95% CI = [0.113, 0.114]) for the baseline model, 0.105 (SD = 0.0033, 95% CI = [0.105, 0.106]) for the 12-month model, 0.0994 (SD = 0.0039, 95% CI = [0.0986, 0.100]) for the 24-month model, and 0.0906 (SD = 0.0034, 95% CI = [0.0899, 0.0913]) for the 36-month model. Longer longitudinal information improves the Brier scores of probabilistic predictions (Kruskal–Wallis *χ*^2^ = 346.1, df = 3, *p* < 2.2e-16).

Figure 2C displays that all models outperformed the two extreme strategies of intervening in all or none of the patients, as indicated by the higher net benefits. Generally, the models with longer longitudinal information had better performance in terms of net benefits. The performance of the models at various thresholds is presented in Table 2 (Supplementary material for examples).

**Table 2:** Performance measures for a range of dichotomous risk score cutoffs.

Model	Threshold	Sensitivity	Specificity	PPV	NPV	NB	sNB
Baseline	0.05	0.99 ± 0.01	0.04 ± 0.05	0.14 ± 0.01	0.97 ± 0.04	0.09 ± 0.00	0.42 ± 0.01
	0.10	0.84 ± 0.06	0.39 ± 0.06	0.18 ± 0.01	0.94 ± 0.02	0.06 ± 0.01	0.26 ± 0.03
	0.15	0.54 ± 0.08	0.69 ± 0.04	0.22 ± 0.03	0.90 ± 0.01	0.03 ± 0.01	0.12 ± 0.04
	0.20	0.33 ± 0.07	0.85 ± 0.03	0.26 ± 0.05	0.89 ± 0.01	0.01 ± 0.01	0.05 ± 0.04
	0.25	0.18 ± 0.06	0.93 ± 0.03	0.29 ± 0.07	0.88 ± 0.01	0.00 ± 0.01	0.02 ± 0.03
	0.30	0.08 ± 0.05	0.97 ± 0.02	0.31 ± 0.15	0.87 ± 0.00	0.00 ± 0.01	0.00 ± 0.02
	0.35	0.03 ± 0.03	0.99 ± 0.01	0.36 ± 0.31	0.87 ± 0.00	0.00 ± 0.00	0.00 ± 0.02
	0.40	0.01 ± 0.01	1.00 ± 0.01	0.31 ± 0.38	0.86 ± 0.00	0.00 ± 0.00	−0.01 ± 0.02
12-month	0.05	0.96 ± 0.04	0.15 ± 0.06	0.14 ± 0.01	0.97 ± 0.03	0.09 ± 0.00	0.39 ± 0.02
	0.10	0.78 ± 0.08	0.49 ± 0.04	0.19 ± 0.01	0.94 ± 0.02	0.05 ± 0.01	0.24 ± 0.04
	0.15	0.55 ± 0.08	0.72 ± 0.03	0.23 ± 0.03	0.92 ± 0.01	0.03 ± 0.01	0.13 ± 0.05
	0.20	0.38 ± 0.06	0.85 ± 0.03	0.28 ± 0.05	0.90 ± 0.01	0.02 ± 0.01	0.08 ± 0.05
	0.25	0.24 ± 0.06	0.93 ± 0.02	0.34 ± 0.08	0.89 ± 0.01	0.01 ± 0.01	0.04 ± 0.04
	0.30	0.15 ± 0.05	0.96 ± 0.01	0.40 ± 0.12	0.88 ± 0.01	0.01 ± 0.01	0.03 ± 0.03
	0.35	0.09 ± 0.04	0.98 ± 0.01	0.45 ± 0.18	0.88 ± 0.00	0.00 ± 0.01	0.01 ± 0.02
	0.40	0.05 ± 0.03	0.99 ± 0.01	0.50 ± 0.27	0.88 ± 0.00	0.00 ± 0.00	0.01 ± 0.02
24-month	0.05	0.97 ± 0.03	0.23 ± 0.05	0.15 ± 0.01	0.98 ± 0.02	0.09 ± 0.00	0.39 ± 0.02
	0.10	0.79 ± 0.07	0.57 ± 0.03	0.21 ± 0.01	0.95 ± 0.01	0.06 ± 0.01	0.26 ± 0.03
	0.15	0.56 ± 0.07	0.77 ± 0.03	0.26 ± 0.03	0.93 ± 0.01	0.03 ± 0.01	0.16 ± 0.04
	0.20	0.40 ± 0.06	0.87 ± 0.02	0.31 ± 0.04	0.91 ± 0.01	0.02 ± 0.01	0.10 ± 0.04
	0.25	0.29 ± 0.06	0.92 ± 0.02	0.35 ± 0.07	0.90 ± 0.01	0.01 ± 0.01	0.06 ± 0.04
	0.30	0.20 ± 0.05	0.95 ± 0.02	0.38 ± 0.09	0.89 ± 0.01	0.01 ± 0.01	0.03 ± 0.04
	0.35	0.14 ± 0.04	0.97 ± 0.01	0.42 ± 0.14	0.89 ± 0.01	0.00 ± 0.01	0.01 ± 0.04
	0.40	0.09 ± 0.04	0.98 ± 0.01	0.45 ± 0.17	0.88 ± 0.00	0.00 ± 0.01	0.00 ± 0.03
36-month	0.05	0.96 ± 0.03	0.32 ± 0.05	0.15 ± 0.01	0.98 ± 0.01	0.08 ± 0.00	0.35 ± 0.01
	0.10	0.74 ± 0.08	0.64 ± 0.03	0.21 ± 0.02	0.95 ± 0.01	0.05 ± 0.01	0.22 ± 0.03
	0.15	0.57 ± 0.07	0.81 ± 0.03	0.28 ± 0.03	0.94 ± 0.01	0.04 ± 0.01	0.16 ± 0.04
	0.20	0.42 ± 0.08	0.89 ± 0.02	0.33 ± 0.05	0.92 ± 0.01	0.02 ± 0.01	0.11 ± 0.04
	0.25	0.31 ± 0.07	0.93 ± 0.02	0.37 ± 0.07	0.91 ± 0.01	0.02 ± 0.01	0.07 ± 0.04
	0.30	0.23 ± 0.06	0.96 ± 0.01	0.41 ± 0.09	0.91 ± 0.01	0.01 ± 0.01	0.04 ± 0.04
	0.35	0.16 ± 0.06	0.97 ± 0.01	0.44 ± 0.11	0.90 ± 0.01	0.01 ± 0.01	0.02 ± 0.03
	0.40	0.10 ± 0.05	0.98 ± 0.01	0.42 ± 0.17	0.89 ± 0.01	0.00 ± 0.01	0.01 ± 0.03

Average feature importance rank of each variable over 100 repeated random subsampling is displayed in Fig. 2D. For the baseline model, the most important features were age at first service contact, schizophrenia diagnosis, age of onset, duration of first episode, days of hospitalization during first episode, days of DUP and DDD at baseline. For the 12-, 24- and 36-month models, longitudinal features, including number of months with relapse (Relapse [sum]), mean DDD, and number of months of anticholinergic use (Anticholinergic [sum]) were the most important features. Mean and MSSD of positive symptoms and SOFAS as well as polypharmacy were also important features.

**Fig. 3:**
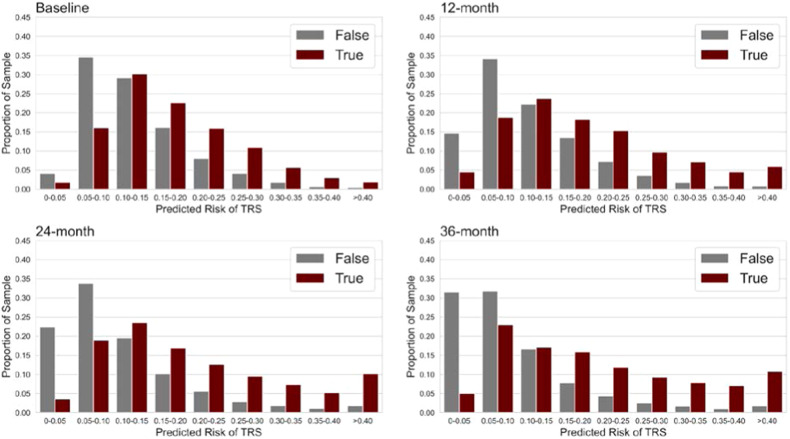
Frequency distributions of predicted risks of an individual whether prescribed with clozapine (true or false) of the baseline, 12-, 24- and 36-month probability classification models. As the predicted risk increases from 0.10 or higher, there is a proportional increase in the number of individuals with clozapine use compared to those without in each subsequent risk class.

Figure 3 presents that patients with higher predicted probability had a higher chance of clozapine use after a threshold of 0.1 for all the models. With a progressively higher predicted probability, the proportion of clozapine use in patients increased. These patterns again suggested that our models were able to differentiate patients with and without future clozapine use.

We evaluated our models with only top 10, top 15, or top 20 features selected by feature importance. Results suggested that models with top 10 features performed similarly in terms of AUROC and Brier score compared to the model with all features (Fig. S1). The baseline model with top 10 features performed slightly better than that with all features. Therefore, the final probability calculator was developed using only top 10 features with all our samples. A description of the calculator program can be found in the supplementary materials.

## Discussion

In this population-based cohort study using intensively collected clinical data over 12 years in Hong Kong, we developed an individualized risk calculator to predict clozapine prescription, a proxy for TR status, using autoML. About 13.7% of FEP patients were prescribed clozapine, similar as in a previous population-based cohort study using Danish registry data (13.2%) [24]. The ML models identified future clozapine users with AUROC ranging from 0.676 (with baseline information) to 0.774 (with 36-month information). The AUROC of models with information across more than 12 months were all over 0.7, suggesting that the models with longitudinal clinical information have an acceptable prediction ability. Models with the top 10 features were found to have similar performance in terms of AUROC and Brier score compared to the full model and were thus used to establish the individualized risk calculator for development of TR in FEP (TRipCal).

Our models performed better than the previous attempts of using machine learning approaches in predicting the development of TR in psychosis [20, 21]. It is likely that the autoML allows for the optimal selection of the machine learning pipelines and hyperparameter optimization, and thus better handles the more complex real-life prediction needs of the psychiatric population. Furthermore, models with longitudinal clinical information performed better. These highlighted that longitudinal clinical information reflecting the dynamics of clinical characteristics and medication treatment over time may be more powerful in predicting the development of TR. The increased use of electronic health records (eHR) and the development of technology such as natural language processing in extracting relevant clinical information from the eHR would allow the automated use of longitudinal clinical information in individual risk calculators. This effort could develop into a data-driven clinical assistant system to support clinicians in tailoring individual patient interventions to postpone or prevent TR development as well as reduce delay in clozapine initiation.

Some of the predictive features identified in the current study are in line with the previous studies [17, 18], such as younger age of onset, schizophrenia diagnosis and relapse [1, 17, 18]. Duration and hospitalization of the first episode as well as the average DDD were found to be prominent features of the baseline prediction model. Having poor response to first-line antipsychotics early in treatment may reflect a different dopamine system function and would be an important indicator in predicting future TR development. This is aligned with findings of neuroimaging studies that patients with TRS have normal dopamine synthesis capacity [36, 37]. The significant role of DDD of antipsychotics and the number of relapses in predicting future TR status suggest the possibility of dopaminergic hypersensitivity as one of the mechanisms of development of TR [16, 38]. One notable finding of the current study is the increasing significance of the use of anticholinergic drugs in predicting future TR status. This may reflect the use of high antipsychotic dose leading to more extrapyramidal side effects, thus more use of anticholinergic medications. On the other hand, the loss of cholinergic neurons has been hypothesized as a possible pathogenesis of tardive dyskinesias, antipsychotic hypersensitivity and refractory status to antipsychotic treatment in patients with schizophrenia in earlier reports [39, 40]. Studies of other cohorts would be needed to replicate these risk factors of TR.

One of the key limitations of the study is the use of clozapine as a proxy for TR. Clozapine may be used to alleviate other conditions such as recurrent suicidality [41] and tardive dyskinesia [42]. However, over 90% of patients who were prescribed with clozapine were considered to have fulfilled the criteria of TRS [1, 43]. Furthermore, there are also individuals who had TR but were not on clozapine, that was estimated to be about 4% in our previous study of similar follow-up duration [1]. This group might impact the performance of the model development. Patients with a wide range of baseline FEP diagnosis were included in this study though 87% of patients on clozapine had a baseline diagnosis of schizophrenia. This approach, though not able to focus specifically on schizophrenia diagnosis and limit the interpretation of the results from a theoretical perspective, may have better translational value as results could be more readily integrated into the current practice of FEP service. Future larger sample studies could focus on the examination of predictors of treatment-resistant schizophrenia, particularly the possible differential predictors of TRS in the first episode and those after multiple episodes. Quality of the data retrieval, particularly the clinical symptoms, depending on the quality of the clinical record, could have contributed to information bias. Third, this study cohort has a limited age range and has a relatively low rate of comorbid substance use. Therefore, results might not be generalizable to other populations, validation studies with cohorts of different countries and characteristics are needed. Finally, a lack of external validation may limit the generalizability of the trained models. Future effort should focus on collecting additional data from diverse sources to validate the model’s performance and ensure its robustness and applicability in real-world scenarios.

In conclusion, our study presented the development of a risk calculator of future clozapine use, a proxy of TR, in FEP patients (TRipCal) over 12–17 years, using both baseline and longitudinal clinical information in the first 36 months of treatment. This work demonstrated the importance of longitudinal clinical information in predicting development of future TR with acceptable accuracy using the AutoML approach and thus the possibility of establishing data-driven tools assisting clinicians for earlier detection of individuals with higher risk of future TR development. The individual calculator developed using the top 10 features identified in the current study could be used to personalize the interventions to prevent, postpone TR development and reduce the delay of clozapine use. Future validation studies in different populations and settings are required.

### Supplementary information

Supplementary methods and results

## Data Availability

Data of the study is available at the reasonable request to the corresponding author for research purpose.

## Code availability

Data was preprocessed in R (version 4.1.3). All the probabilistic classification models were developed in Python (version 3.8.13) with scikit-learn and TPOT modules. Visualization was done in Python using the matplotlib module and R using the ggplot2 package. The calculator program was implemented using the tinker module in Python. All our analysis scripts are available at https://github.com/kamione/clozapineuse_prediction and source codes of our probability calculator application can be found at https://github.com/kamione/prob_calculator_clozapine.
