# Supplementary material for: Development of an individualized risk calculator of treatment resistance in patients with first-episode psychosis (TRipCal) using automated machine learning: a 12-year follow-up study with clozapine prescription as a proxy indicator
Source: Transl Psychiatry. 2024 Jan 22;14:50. doi: 10.1038/s41398-024-02754-w (PMC10803337; doi:10.1038/s41398-024-02754-w)
Supplement: Supplementary file 1 — Supplementary methods and results [file 41398_2024_2754_MOESM1_ESM.docx]

**Development of an individualized risk calculator of treatment resistance in patients with first-episode psychosis (TRipCal) using automated machine learning: A 12-year follow-up study with clozapine prescription as a proxy indicator**

**Supplementary Materials**

**Methods**

**Operational definition of the features and quality assurance of data:**

The diagnosis of patients was determined by clinicians based on the International Statistical Classification of Diseases and Related Health Problems (10th edition) criteria. DUP was defined as the duration (in days) between first psychotic symptom emergency and the use of effective psychiatric treatment as determined by clinicians. Relapse was operationally defined as a >=2 point increase of CGI-SCH positive scores (e.g., from 2 to 4) followed with hospitalization or adjustment of antipsychotic medication. Medication adherence was evaluated for each patient using a score of 1-3, with 1 indicating good adherence and 3 indicating poor adherence. Polypharmacy scores from 0 to 2, with 0 indicating no medication, 1 indicating only 1 medication and 2 indicating 2 or more medications. The clinical assessments were conducted by a clinical research team consisting of psychiatrists and research assistances. To assure the data quality, research assistants met with the clinicians every 2 weeks during the data collection period to achieve consensus. We evaluated interrater reliability for the major variables using the records of 12 patients. A satisfactory level of concordance was revealed using the intraclass correlation coefficient (ICC) between clinicians and research staff (DUP: ICC = 0.78, CGI- SCH positive: ICC = 0.89, and CGI-SCH negative: ICC = 0.77).

**Ethics Approval Details:**

The current study has been approved by 6 ethics committee of 7 hospital cluster with details as followings: Kowloon West Cluster Institution Review Board (Reference number: KW/FR-14-168(79-06)), Kowloon Central and Kowloon East Institution Review Board (Reference number: KC/KE-14-0163/ER-3), Hong Kong East Cluster Institution Review Board (Reference number: HKEC-2014-097), HKU/Hong Kong West Cluster Institution Review Board (Reference number UW 12 – 522), New Territory East Cluster Institution Review Board (Reference number NTEC 2014.656), New Territory West Cluster Institution Review Board (Reference number NTWC/CREC/1351/14).

**Decision curve analysis**

In the decision curve analysis, the performance of an event predictor is evaluated by varying the probability threshold and plotting a graphical representation of net benefit against the threshold probability. To calculate the individual net benefit at a given threshold (*pt*), the following formula was used 1:


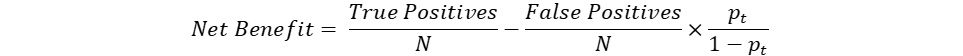


Here, N represents the total sample size, *pt* represents the threshold probability used to define

when a patient is positive, True Positives represent the number of patients correctly identified as positive, and False Positives represent the number of patients incorrectly identified as positive. The decision curves for the "intervention for all" and "intervention for none" strategies were included because they are considered reasonable clinical approaches 2.

**Results**

Examples for the decision curve analysis as supplement for Table 2. For instance, using a 12- month model with a less strict cutoff of 0.15 would detect 55% of positive cases, with 23% of selected individuals expected to use clozapine within 11 years. At this cutoff, the model detected a further 13% of future clozapine use cases based on the standardized net benefits with an estimated prevalence of 22%. Conversely, a 36-month model with a more stringent cutoff of 0.25 would identify 31% of positive cases, and 37% of them would be expected to use clozapine within 9 years, leading to an additional 7% detection of future clozapine use cases.

**Individual Risk Calculator**

The individual risk calculator is developed using Python (version 3.8.13). Details of how to create an environment and deploy the application can be found in https://github.com/kamione/prob_calculator_clozapine. The application will initiate a data-

entry interface (as shown in the figure below), allowing users to enter information for predicting the risk. The tabs on the top left allows users to enter information with different lengths of longitudinal information (i.e., baseline, 12-month, 24-month and 36-month).


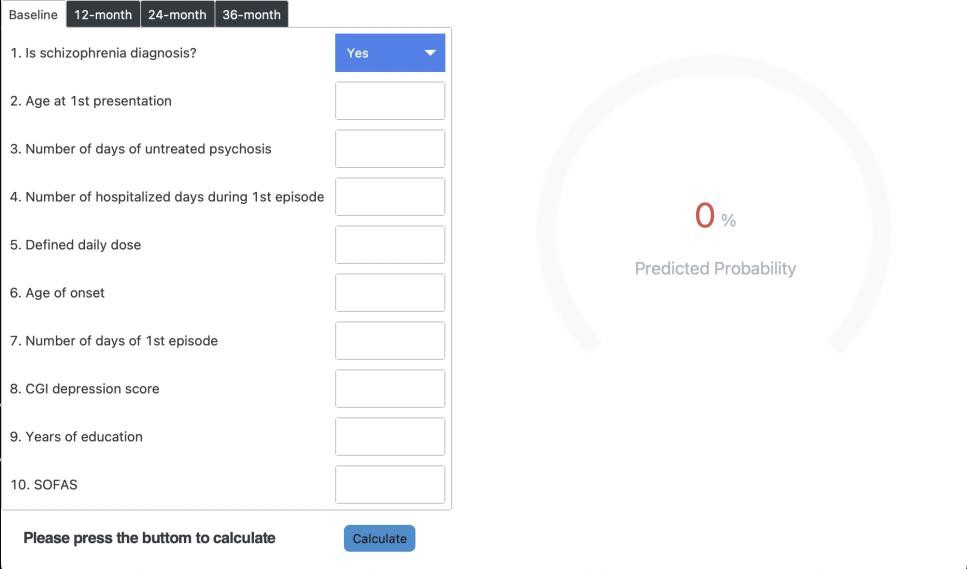


Here we demonstrated the app using a 36-month tab. First, the user can enter all the required information. Any incorrect data type will raise a warning window and require users to re- enter correct information. After all the cells are filled, the user can press the calculate button to calculate the predicted probability of a particular patient using our pretrained model. The predicted probability will be shown on the right panel.


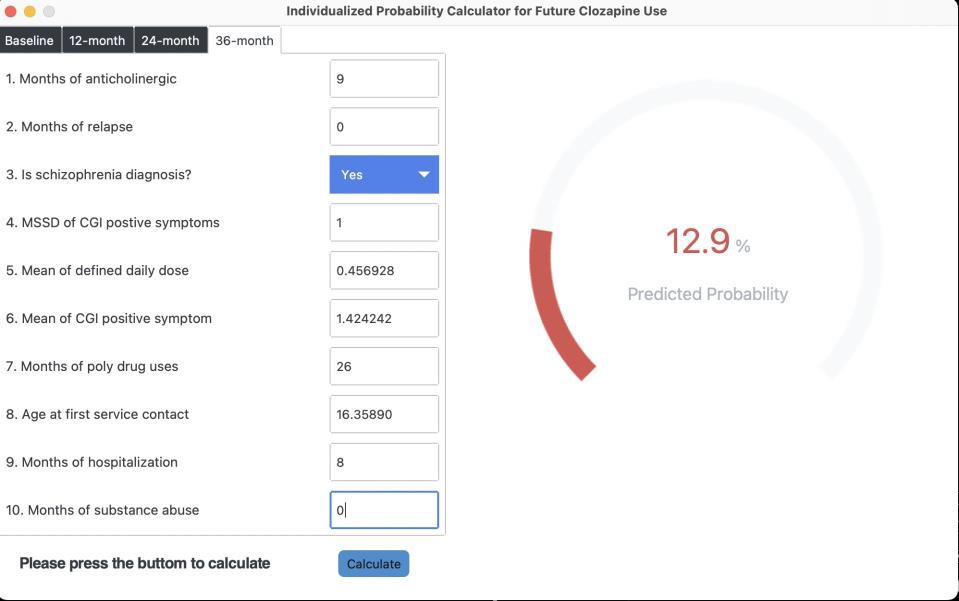


# Table S1. Checklist from Transparent Reporting of a multivariable prediction model for Individual Prognosis Or Diagnosis (TRIPOD) guidelines

| **Section/Topic** | **Item** | **Development or validation** | **Checklist item** | **Page** |
| --- | --- | --- | --- | --- |
| **Title and Abstract** |  |  |  |  |
| **Title** | **1** | **D;V** | **Identify the study as developing and/or validating a multivariable prediction model, the target population, and the outcome to be predicted.** | **1** |
| **Abstract** | **2** | **D;V** | **Provide a summary of objectives, study design, setting, participants, sample size, predictors, outcome, statistical analysis, results, and conclusions.** | **1-3** |
| **Introduction** |  |  |  |  |
| **Background and objectives** | **3a** | **D;V** | **Explain the medical context (including whether diagnostic or prognostic) and rationale for developing or validating the multivariable prediction model, including references to existing models.** | **4-5** |
|  | **3b** | **D;V** | **Specify the objectives, including whether the study describes the development or validation of the model, or both.** | **5** |
| **Methods** |  |  |  |  |
| **Source of data** | **4a** | **D;V** | **Describe the study design or source of data (e.g., randomized trial, cohort, or registry data), separately for the development and validation data sets, if applicable.** | **5-6** |
|  | **4b** | **D;V** | **Specify the key study dates, including start of accrual; end of accrual; and, if applicable, end of follow-up.** | **5-6** |
| **Participants** | **5a** | **D;V** | **Specify key elements of the study setting (e.g., primary care, secondary care, general population) including number and location of centres.** | **5-6** |
|  | **5b** | **D;V** | **Describe eligibility criteria for participants.** | **5-6** |
|  | **5c** | **D;V** | **Give details of treatments received, if relevant.** | **NR** |
| **Outcomes** | **6a** | **D;V** | **Clearly define the outcome that is predicted by the prediction model, including how and when assessed.** | **5-6** |
|  | **6b** | **D;V** | **Report any actions to blind assessment of the outcome to be predicted.** | **NR** |
| **Predictors** | **7a** | **D;V** | **Clearly define all predictors used in developing the multivariable prediction model, including how and when they were measured.** | **6-7** |
|  | **7b** | **D;V** | **Report any actions to blind assessment of predictors for the outcome and other predictors.** | **Supple menta ry** |

| **Sample size** | **8** | **D;V** | **Explain how the study size was arrived at.** | **5** |
| --- | --- | --- | --- | --- |
| **Missing data** | **9** | **D;V** | **Describe how missing data were handled (e.g., complete-case analysis, single imputation, multiple imputation) with details of any imputation method.** | **7** |
| **Statistical analysis methods** | **10a** | **D** | **Describe how predictors were handled in the analyses.** | **7-8** |
|  | **10b** | **D** | **Specify type of model, all model building procedures (including any predictor selection), and method for internal validation.** | **7-8** |
|  | **10c** | **V** | **For validation, describe how the predictions were calculated.** | **7-8** |
|  | **10d** | **D;V** | **Specify all measures used to assess model performance and, if relevant, to compare multiple models.** | **7-8**  **Supple menta ry** |
|  | **10e** | **V** | **Describe any model updating (e.g., recalibration) arising from the validation, if done.** | **7-8**  **Supple menta ry** |
| **Risk groups** | **11** | **D;V** | **Provide details on how risk groups were created, if done.** | **NR** |
| **Development vs validation** | **12** | **V** | **For validation, identify any differences from the development data in setting, eligibility criteria, outcome, and predictors.** | **7-8** |
| **Results** |  |  |  |  |
| **Participants** | **13a** | **D;V** | **Describe the flow of participants through the study, including the number of participants with and without the outcome and, if applicable, a summary of the follow-up time. A diagram may be helpful.** | **9** |
|  | **13b** | **D;V** | **Describe the characteristics of the participants (basic demographics, clinical features, available predictors), including the number of participants with missing data for predictors and outcome.** | **9**  **Table 1** |
|  | **13c** | **V** | **For validation, show a comparison with the development data of the distribution of important variables (demographics, predictors and outcome).** | **9-10** |
| **Model development** | **14a** | **D** | **Specify the number of participants and outcome events in each analysis.** | **8, 9-10** |
|  | **14b** | **D** | **If done, report the unadjusted association between each candidate predictor and outcome.** | **NR** |
| **Model specification** | **15a** | **D** | **Present the full prediction model to allow predictions for individuals (i.e., all regression coefficients, and model intercept or baseline survival at a given time point).** | **9-10** |
|  | **15b** | **D** | **Explain how to use the prediction model.** | **9-10** |

| **Model performance** | **16** | **D;V** | **Report performance measures (with CIs) for the prediction model.** | **Table 2** |
| --- | --- | --- | --- | --- |
| **Model updating** | **17** | **V** | **If done, report the results from any model updating (i.e., model specification, model performance).** | **Supple menta ry** |
| **Discussion** |  |  |  |  |
| **Limitations** | **18** | **D;V** | **Discuss any limitations of the study (such as nonrepresentative sample, few events per predictor, missing data).** | **12** |
| **Interpretation** | **19a** | **V** | **For validation, discuss the results with reference to performance in the development data, and any other validation data.** | **11-12** |
|  | **19b** | **D;V** | **Give an overall interpretation of the results, considering objectives, limitations, results from similar studies, and other relevant evidence.** | **11-12** |
| **Implications** | **20** | **D;V** | **Discuss the potential clinical use of the model and implications for future research.** | **13** |
| **Other informatio** | **n** |  |  |  |
| **Supplementary information** | **21** | **D;V** | **Provide information about the availability of supplementary resources, such as study protocol, Web calculator, and data sets.** | **Supple menta ry** |
| **Funding** | **22** | **D;V** | **Give the source of funding and the role of the funders for the present study.** | **14** |
| ***Items relevant only to the development of a prediction model are denoted by D, items relating solely to a validation of a prediction model are denoted by V, and items relating to both are denoted D;V. We recommend using the TRIPOD Checklist in conjunction with the TRIPOD explanation and elaboration document.** | | | | |

**Figure S1. Comparing models with all features to models with only top 10, 15, or 20 features.** Models with different numbers of features performed similarly in general in terms of

1. AUROC and B. Brier scores. Although some significant differences were detected across models (uncorrected *p* values < 0.05), the models with Top 10 performed as good as others.


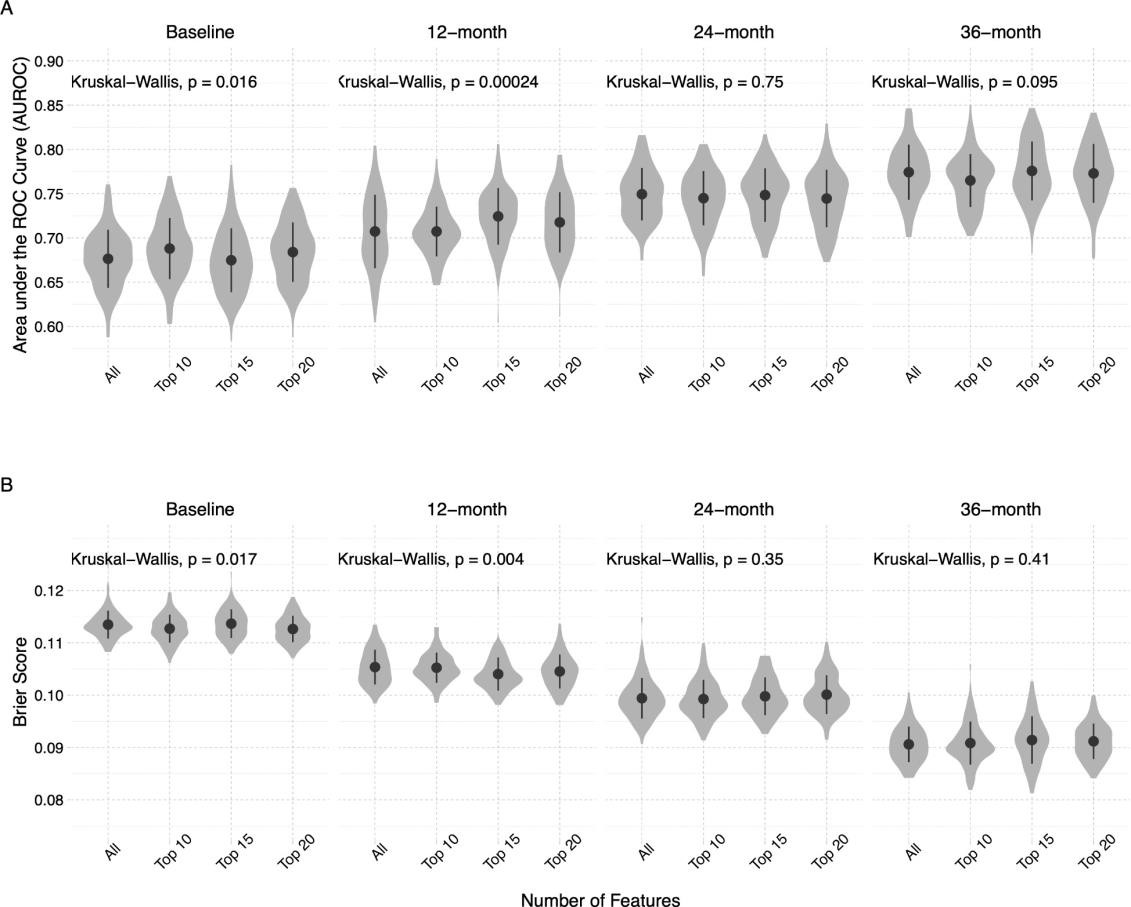


References

- 1. Vickers AJ, Van Calster B, Steyerberg EW. Net benefit approaches to the evaluation of prediction models, molecular markers, and diagnostic tests. *BMJ*. 2016;352:i6.
  2. Pauker SG, Kassirer JP. The threshold approach to clinical decision making. *N Engl J Med*. 1980;302(20):1109-1117.
